# Time-effective analgesic effect of acupressure ankle strip pressing wrist and ankle acupuncture point on primary dysmenorrhea: Study protocol clinical trial (SPIRIT compliant)

**DOI:** 10.1097/MD.0000000000019496

**Published:** 2020-03-20

**Authors:** Shu-jie Zhai, Yi Ruan, Yue Liu, Zhen Lin, Chen Xia, Fan-fu Fang, Qing-hui Zhou

**Affiliations:** a School of Acupuncture-Moxibustion and Tuina, Shanghai University of Traditional Chinese Medicine; b School of Traditional Chinese Medicine, Naval Medical University, Shanghai, China; c Department of Health Statistics, Naval Medical University; d Department of Rehabilitation Medicine, Changhai Hospital, Shanghai, China.

**Keywords:** wrist-ankle acupuncture, primary dysmenorrhea, acupressure wrist-ankle strap, randomized controlled trial, study protocol

## Abstract

**Background:**

Dysmenorrhea seriously affects the ability of women to perform normal social activities and decreases their quality of life. Primary dysmenorrhea can be effectively treated with acupuncture. Based on the wrist-ankle acupuncture (WAA) theory, we designed a portable WAA point compression treatment strap that treats diseases by automatically applying pressure to acupuncture points. The proposed study aims to evaluate the immediate analgesic effect of the acupressure wrist-ankle strap in patients with primary dysmenorrhea.

**Methods:**

The study will be a randomized controlled trial conducted from May 1, 2019 to May 30, 2020 that includes 78 students from Shanghai University of Traditional Chinese Medicine who have primary dysmenorrhea and meet the eligibility criteria. Participants will be randomly divided into 2 groups in a 1:1 allocation ratio. The intervention group will use the acupressure wrist-ankle strap equipped with tip compression component parts on the internal side; the control group will use the nonacupressure wrist-ankle strap with the tip compression parts removed. All participants will be treated for 30 minutes on the 1st day of menstruation. The primary outcome is the pain intensity score measured by the visual analog scale. The secondary outcomes are the onset time of analgesia, the pain threshold at Yinlingquan (SP 9), skin temperature at Guanyuan (CV 4), and expectations and satisfaction of patients as investigated via the expectation and treatment credibility scale.

**Discussion:**

This trial will be the 1st study to evaluate the analgesic effect of the acupressure wrist-ankle strap in patients with primary dysmenorrhea. The quality of this study is ensured by the randomization, nonacupressure control, and blinded design. The results may provide evidence for a potential alternative treatment for primary dysmenorrhea and evidence-based proof of the analgesic effect of WAA.

## 1 Introduction

Dysmenorrhea is a condition in which pain is experienced in the lower abdomen and lumbosacral area during menstruation. Patients with severe dysmenorrhea have associated nausea, vomiting, diarrhea, and even syncope.^[1]^ Primary dysmenorrhea is more common in unmarried women, as they typically have no organic lesions in the reproductive organs; primary dysmenorrheic pain is mainly caused by uterine muscle spasm or tonic contraction. Secondary dysmenorrhea mainly prevails in married people, mostly due to pelvic inflammation, uterine fibroids, endometriosis, and other diseases.^[2]^ Because of differences in the definition of dysmenorrhea and/or the methods used to measure dysmenorrheic pain, the prevalence of dysmenorrhea varies widely between countries, but generally ranges from 50% to 90%.^[3]^ Primary dysmenorrhea not only causes the abovementioned symptoms, but can also be associated with diseases such as interstitial cystitis, arrhythmia, and irritable bowel syndrome, which seriously affect normal social activities, quality of life, and increase the mental and psychological burden of patients.^[4]^ Furthermore, dysmenorrheic patients may be unable to attend work and school, which has an impact on social economy and education.^[5,6]^ Previous studies reported that 25% to 51% of dysmenorrheic patients are unable to study, work, and live normally.^[7,8]^ Therefore, it is important to investigate ways to reduce the labor loss caused by primary dysmenorrhea and improve the quality of life of patients.

In western medicine, the symptoms of primary dysmenorrhea are treated or controlled using analgesics, nonsteroidal anti-inflammatory drugs, and oral contraceptives. However, these drugs may trigger gastrointestinal reactions and/or affect the central nervous system and metabolism, leading to poor long-term effects, drug resistance, and adverse effects.^[9]^

Acupuncture therapy plays an important role in the clinical treatment of primary dysmenorrhea^[10]^ due to its safety, rapid onset of analgesic effect, low cost, and low incidence of adverse effects.^[11]^ Wrist-ankle acupuncture (WAA) is more readily accepted by patients than traditional acupuncture and electroacupuncture, as WAA is easy to operate and causes very minimal pain and discomfort during the implementation process.^[12]^ A systematic review of WAA for the treatment of pain showed that WAA alone and WAA as an adjuvant therapy were more effective than Western medicine, sham acupuncture, or body acupuncture, with few adverse effects.^[13]^ A series of studies conducted by our research group also showed that WAA has a good analgesic effect,^[14–21]^ and the analgesic effect of WAA for acute low-back pain is immediate.^[18]^ However, WAA requires the operator to master specific techniques and thus needs to be performed by acupuncturists. Hence, our research team developed the acupressure wrist-ankle strap, which is a new portable WAA point compression treatment device^[22]^ that aims to replace the need for acupuncture needles and enable the patients to operate it themselves to obtain pain relief.

Numerous studies have proved that WAA significantly reduces pain in patients with primary dysmenorrhea.^[23–26]^ Our hypothesis is that the acupressure wrist-ankle strap, which stimulates the WAA stimulation, will also produce an immediate analgesic effect in patients with dysmenorrhea, a condition with acute pain. Thus, we designed the randomized controlled trial (RCT) described in this study protocol to evaluate the immediate analgesic effect of the acupressure wrist-ankle strap in patients with primary dysmenorrhea.

## 2 Methods and design

### 2.1 Objectives

The proposed study has 3 objectives: to determine whether the acupressure wrist-ankle strap has an immediate analgesic effect in patients with primary dysmenorrhea; to explore the possible antidysmenorrheic mechanism of the acupressure wrist-ankle strap; and to explore the factors potentially affecting the therapeutic effect of the acupressure wrist-ankle strap.

### 2.2 Trial design

This study is an RCT designed to compare the immediate analgesic effect of the acupressure wrist-ankle strap vs a nonacupressure wrist-ankle strap in the treatment of primary dysmenorrhea. Participants with primary dysmenorrhea will be randomly divided into 2 groups in a 1:1 ratio.

### 2.3 Participants

#### 2.3.1 Inclusion criteria

The inclusion criteria are: women aged 18 to 30 years; meeting the criteria for the diagnosis of primary dysmenorrhea based on the Primary Dysmenorrhea Consensus Guidelines^[27]^; basic regularity of the menstrual cycle (28 ± 7 days); no ongoing treatment for dysmenorrhea; provision of written informed consent; and mean pain intensity score evaluated by the visual analog scale (VAS) of >2 for 3 consecutive menstrual cycles.

#### 2.3.2 Exclusion criteria

The exclusion criteria are: pregnancy, prepregnancy, or lactation; having experienced miscarriage or stillbirth; cardiovascular, hepatic, renal, hematopoietic, and other serious primary diseases or mental disorders; being suspected of having other diseases based on the medical history; having taken any analgesic medications within 24 hours before the intervention.

#### 2.3.3 Recruitment

The recruitment targets are undergraduate and graduate students at Shanghai University of Traditional Chinese Medicine. Recruitment posters will be put up on campus.

#### 2.3.4 Patient safety

The acupressure wrist-ankle strap should not be worn for too long. It is recommended to take it off after wearing for about 30 minutes to prevent the local blood flow from obstruction and avoid other risk factors. If the participants are still in severe pain after 30-minute treatment with the acupressure wrist-ankle strap, a variety of follow-up treatments will be provided, including traditional acupuncture, moxibustion, and analgesic medications. Participants with continuous high pain after the above treatment will be referred to a general physician or gynecologist to receive other form of treatment.

### 2.4 Intervention

All participants will go through a standardized interview in which the details of the study will be explained. All treatments will be performed by the same registered acupuncturist. During the treatment, the subject will be in a supine position. The acupuncturist is responsible for setting up the treatment device, and putting it in place for the participants. The details of treatment will be fully documented in accordance with the CONSORT Statement and the Standards for Reporting Interventions in Controlled Trials of Acupuncture (STRICTA).^[28]^

#### 2.4.1 Brief introduction to the portable WAA point compression treatment device

**Figure 1:**
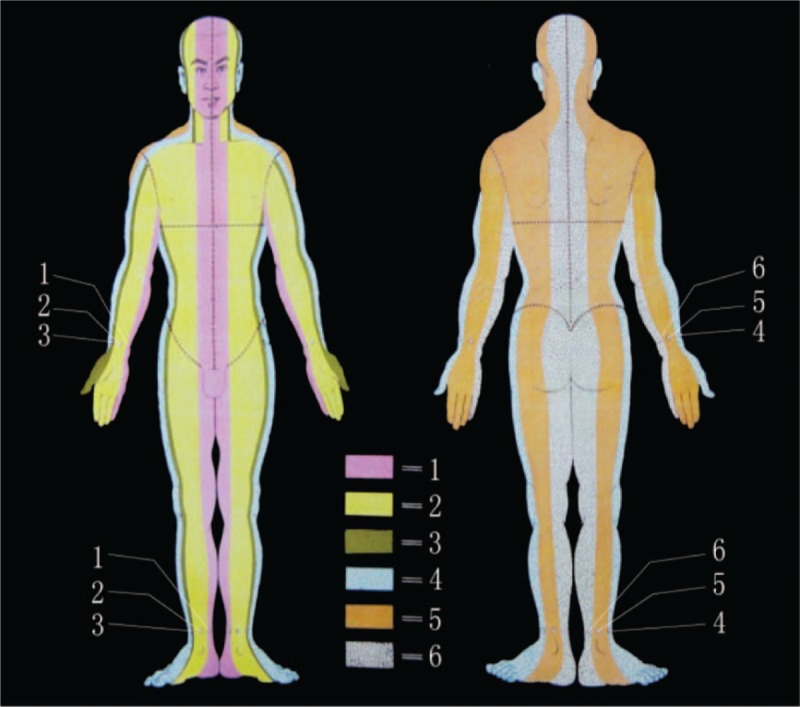
Wrist-ankle acupuncture zones and needling points. Each side of the body is divided into 6 vertical zones numbered 1 to 6, and a circular horizontal line is drawn at the level of the diaphragm to divide the body into upper and lower halves. One acupuncture point is defined at the wrist or ankle in each vertical zone. The name of the acupuncture point is the same as that of the corresponding vertical zone. Acupuncture points at the wrist are used to treat pain in the upper body, while acupuncture points at the ankle are used to treat pain in the lower body. The acupuncture point selected for treatment is that with the same numbered name that corresponds to the vertical zone where the pain is located.

In accordance with the basic principles of WAA therapy, each side of the body is divided into 6 vertical zones, and 1 acupuncture point is defined at the wrist and ankle in each vertical zone. The name of the acupuncture point is the same as that of the corresponding vertical zone. A circular horizontal line is drawn at the level of the diaphragm to divide the body into upper and lower halves. Acupuncture points at the wrist are used to treat pain in the upper body, while acupuncture points at the ankle are used to treat pain in the lower body. The acupuncture point selected for treatment is that with the same numbered name of the corresponding vertical zone where the pain is located^[12]^ (Fig. 1).

**Figure 2:**
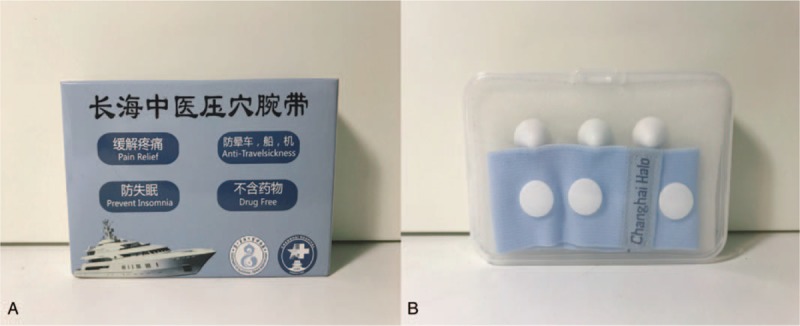
Acupressure wrist-ankle strap as the finished product. (A) The product packaging. (B) The product.

The portable WAA point compression treatment device,^[22]^ the acupressure wrist-ankle strap, is an instrument developed based on WAA therapy that is used instead of invasive acupuncture treatment (Fig. 2). The wrist-ankle strap has removable compression parts that exert pressure and stimulate the corresponding WAA points (compression points). The wrist-ankle strap is equipped with installation bases for 6 fixed compression parts, corresponding to the positions of 6 compression points on the wrist and ankle. Before being worn by the patient, 1 to 2 compression parts are installed in accordance with the individual acupuncture needs of the patient. When the acupressure wrist-ankle strap is being worn, the tips of the compression parts are located at the compression points. For treatment, compression points upper 1 to upper 6 on the wrist and lower 1 to lower 6 on the ankle will be selected, corresponding to the zones where the pain is identified, in accordance with the theory of WAA. Compression of different wrist-ankle compression points relieves pain located on the corresponding zones.

#### 2.4.2 Acupressure wrist-ankle strap group (intervention group)

**Figure 3:**
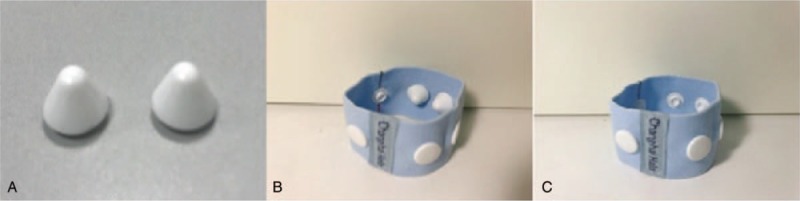
Parts of the acupressure wrist-ankle strap. (A) The removable compression parts. (B) Wrist-ankle strap with the compression parts installed. (C) Wrist-ankle strap with the compression parts removed.

**Figure 4:**
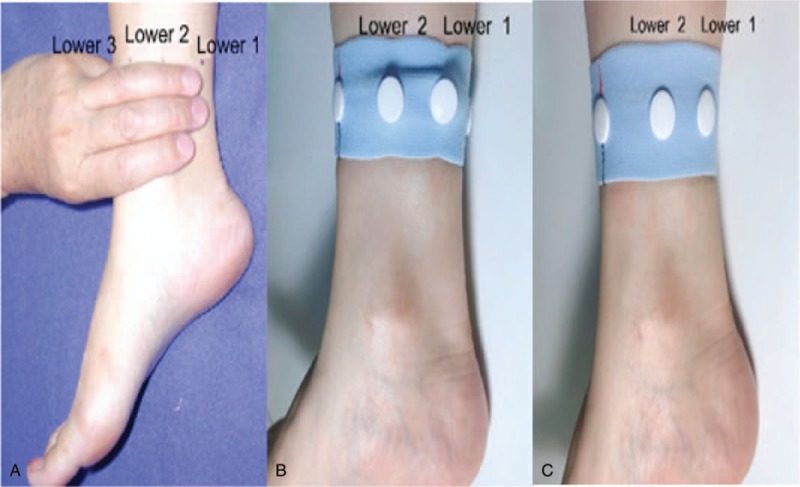
Application of the wrist-ankle strap to the lower 1 and lower 2 regions. Photographs show (A) unilateral locations of the lower 1 and lower 2 regions, (B) unilateral compressed wrist-ankle strap, and (C) unilateral noncompressed wrist-ankle strap.

Abdominal pain in patients with primary dysmenorrhea is located on the lower 1 and lower 2 zones. Thus, the bilateral lower 1 and lower 2 acupuncture points will be stimulated. The lower 1 point is located at the medial border of the calcaneus tendon, while the lower 2 point is located at the posterior border of the humerus, both at the level 3 inches above the tip of the medial malleolus. The acupressure wrist-ankle strap will be used to replace the stimulation achieved with acupuncture needles. Two compression parts will be installed inside the wrist-ankle strap for each patient (Fig. 3). Patients will wear a wrist-ankle strap on both sides for 30 minutes, so that both the lower 1 and lower 2 compression points are simultaneously pressed (Fig. 4).

#### 2.4.3 Nonacupressure wrist-ankle strap group (control group)

Patients will wear a wrist-ankle strap on both sides for 30 minutes as the intervention group, but the compression parts will not be installed on the inside of the straps (Figs. 3 and 4).

### 2.5 Outcome measures

#### 2.5.1 Primary outcome measures

The primary outcome measure is the abdominal pain intensity score assessed by VAS. Abdominal pain will be quantified using the VAS score as determined on a 100-mm horizontal line (0, no pain; 100, pain intolerable). On the VAS scale, the score is based on the horizontal distance from the leftmost pain free to the edge marked by the patient. The distance represents pain intensity. The VAS will be assessed at baseline (3 minutes before intervention) and at 5, 10, and 30 minutes (after the removal of the wrist-ankle strap).

#### 2.5.2 Secondary outcome measures

##### 2.5.2.1 Onset time of analgesia

The analgesic onset time is defined as the time taken for the dysmenorrheic pain to be significantly relieved as reported by the participants.

##### 2.5.2.2 Pain threshold measurement

The pain threshold will be measured at the Yinlingquan acupoint (SP 9) using a pressure pain detector (YISIDA, Hongkong, China). Measurements will be made 3 minutes before treatment and at the end of treatment (after the acupressure wrist-ankle strap is removed).

##### 2.5.2.3 Skin temperature at Guanyuan acupoint (CV 4)

The skin temperature at Guanyuan (CV 4) will be measured using an infrared thermal imager (FLIR ONE, FLIR System, Wilson, Oregon, USA). Measurements will be made 3 minutes before treatment, at the analgesic onset time, and at the end of treatment (after the acupressure wrist-ankle strap is removed).

##### 2.5.2.4 Patient expectations and satisfaction with outcomes

Patient expectation and satisfaction with the curative effect will be investigated using the expectation and treatment credibility scale (ETCS).^[29]^ The ETCS includes 4 statements with a yes/no response: ETCS1: I am sure this treatment will relieve my pain; ETCS2: I think this treatment is reasonable; ETCS3: I will recommend this therapy to my friends; ETCS4: I believe this therapy can cure other diseases. Both groups will complete the ETCS questionnaire 3 minutes before treatment and 3 minutes after the 30-minute treatment.

### 2.6 Study procedure

**Figure 5:**
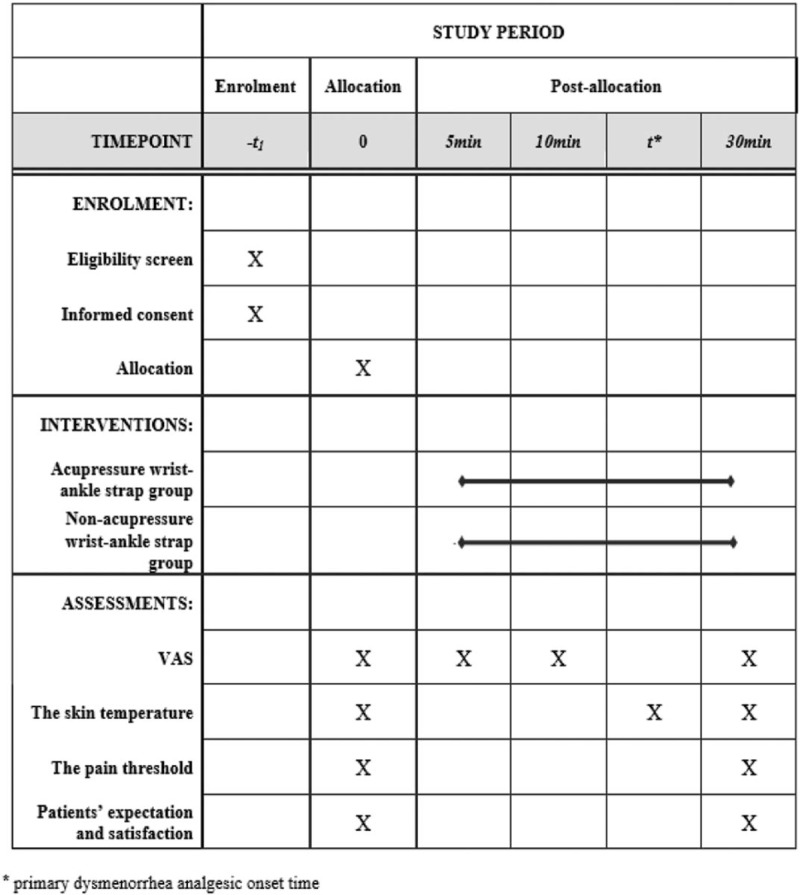
The schedule of enrolments, allocation, and assessments.

**Figure 6:**
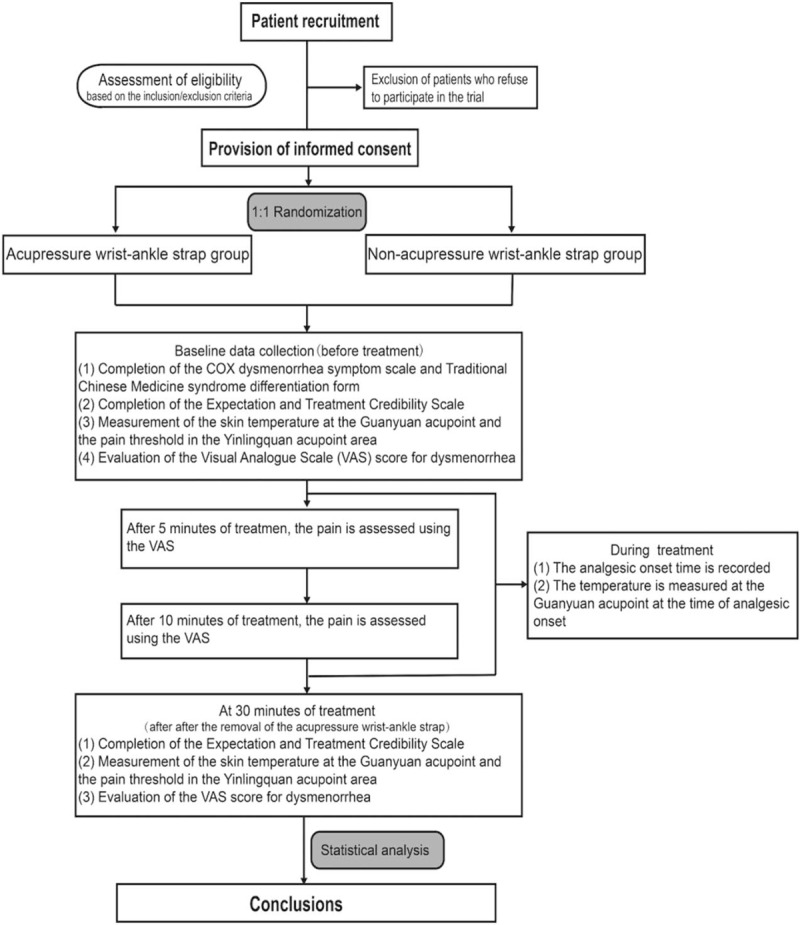
Flow diagram of the proposed study.

Baseline demographic and clinical characteristics will be recorded before study commencement. Participants will complete a general information form, the Cox menstrual symptom scale (CMSS),^[30]^ the traditional Chinese medicine (TCM) syndrome differentiation form,^[31]^ and provide written informed consent. Subjects who meet the eligibility criteria will be enrolled, grouped, and given corresponding interventions. The schedule of enrolments, allocation and assessments is given in Figure 5. The participant flow diagram is illustrated in Figure 6.

### 2.7 Sample size calculation

The participants will be randomly divided into the acupressure wrist-ankle strap group (intervention group) and the nonacupressure wrist-ankle strap group (control group) in a 1:1 allocation ratio. Sample size calculation is based on the primary outcome, which is proposed to be the VAS. PASS 11 software (NCSS, Kaysville, UT) was used to calculate the required sample size. Based on the preliminary experiments, the mean baseline VAS was 74 mm with a standard deviation (*σ*) of 14 mm. The researchers expect the mean difference in the VAS score between the 2 groups (*D*1) will be 10 mm and the correlation between measurement points (*ρ*) will be 0.7, which is not moderately clinically meaningful but represents a minimally important difference. With the test levels set at *α* = 0.05 (2-sided), *β* = 0.1, *D*1 = 1, *M* = 4, *σ* = 1.4, *ρ* = 0.7, and the module selected as “Tests for Two Means in a Repeated Measures Design,” where *α* represents the probability of rejecting a true null hypothesis, *β* is the probability of obtaining a false negative with the statistical test, and *M* is the number of repeated measurements. Finally, at least 32 participants are required per group. Considering a 20% dropout rate, 39 participants will be enrolled in each group, giving a total of 78 participants.

### 2.8 Randomization and blinding procedures

Random numbers will be generated by a computerized random number table generator using the stratified block randomization method of the SAS package (SAS statistical software version 9.4; SAS Institute Inc, Cary, NC). The random number table file will be password-protected and will be managed by an independent, blinded statistician who is not involved in participant recruitment, treatment, or assessment. Eligible subjects who provide written informed consent will be randomly assigned to a group. The participants and the researchers who evaluate the results will be unaware of which intervention group the participants have been assigned to. The wrist-ankle straps used in both groups will have the same packaging. As only one 30-minute treatment will be given, the participants will not be aware of the differences between the products during the treatment.

### 2.9 Data collection and management

The CMSS and the TCM syndrome differentiation form will be used to assess the symptoms of primary dysmenorrhea in the current menstrual cycle before treatment, while the ETCS will be completed before and after the treatment. Participants will complete the general information questionnaire 3 minutes before the treatment. Researchers will ensure the reliability and validity of the questionnaire by providing guidance for each item. Clinical observation results will be recorded on a uniformly printed and numbered clinical observation form. A corresponding computer database will be established and the data will be inputted into the computer on the day of observation.

### 2.10 Statistical analysis

The SPSS for Windows 21.0 will be used for statistical processing. All analyses will be conducted in accordance with the intent-to-treat principle and the per-protocol principle. Measurement data will be expressed as mean ± standard deviation, while count data will be described as number and percentage. The normal distribution test and homogeneity test of variance will be used for measurement data. The value of missing data will be defined based on the last-observation-carried-forward principle. The baseline characteristics of the 2 groups will be analyzed using the *t* test or nonparametric test. If the baseline characteristics are imbalanced, covariance analysis is used to adjust the baseline characteristics. Repeated measures analysis of variance will be adopted to analyze the outcome (analgesic effect) between groups and observed time. If necessary, a mixed model will be used to evaluate the analgesic effect. A general linear model analysis will be used to assess the correlation between patient expectations and the effect. All statistical tests will be performed on both sides, and the difference will be considered statistically significant when *P* < .05.

### 2.11 Ethics and trial registration

The trial protocol is in accordance with the principles of the Declaration of Helsinki^[32]^ and was approved by the China Ethics Committee of Registering Clinical Trials (Ethics Reference: ChiECRCT20190037). This trial was registered in the Chinese Clinical Trial Registry on March 7, 2019 (ID: ChiCTR1900021727). Written informed consent will be obtained from each participant before enrollment.

## 3 Discussion

The WAA is a unique kind of acupuncture, as the needling points are selected in accordance with body divisions and participants do not need to achieve the sensation of de qi, which is usually experienced as sourness, distention, and pain in traditional acupuncture.^[33]^ Acupuncture at the bilateral lower 1 and lower 2 points reportedly achieves a total effective rate of 96.43% in the treatment of primary dysmenorrhea, and WAA achieves a significant rapid reduction in the pain associated with primary dysmenorrhea.^[34]^

Based on the theory of WAA, the pain caused by primary dysmenorrhea is located in the lower 1 or lower 2 areas, and so we chose lower 1 and lower 2 as the points for intervention. The needling points of WAA are distributed on the meridians; lower 1 is on the Foot Shaoyin Kidney Channel, while lower 2 is on the Foot Taiyin Spleen Channel.^[35,36]^ The position of the lower 2 needling point is just at Sanyinjiao (SP 6), which is the intersecting point of the 3 yin channels of the foot, and connects with the uterus through channels and collaterals. SP 6 is one of the best acupoints with immediate analgesic effect for dysmenorrhea.^[37]^ Therefore, the theory of WAA provides a theoretical basis for the ability of the acupressure wrist-ankle strap to alleviate the pain of primary dysmenorrhea.

Primary dysmenorrhea has a high incidence.^[38]^ During the onset of dysmenorrhea, patients often cannot access a doctor due to the severity of symptoms, and patients themselves often use few analgesic approaches. Furthermore, an unhealthy lifestyle, lack of self-care awareness, and chronic recurrent menstrual pain may cause a fear of menstruation and produce anxiety, which aggravates the symptoms of dysmenorrhea. Thus, there is a need for a physical intervention method that can be used to alleviate pain at any time and place.

The purpose of this proposed RCT is to evaluate the immediate effect of the acupressure wrist-ankle strap in relieving pain caused by primary dysmenorrhea. The immediate analgesic effect of this acupressure wrist-ankle strap will be determined based on VAS scores, and the onset time of dysmenorrheic pain relief. The possible mechanisms of the acupressure wrist-ankle strap will be investigated by recording and observing the change in the pain threshold at Yinlingquan (SP 9), and the temperature change in the Guanyuan (CV 4) area. The CMSS and TCM syndrome scale will be used to analyze the correlation between the analgesic effect of the acupressure wrist-ankle strap and the TCM syndrome and severity of the disease.

The acupressure wrist-ankle strap is a physical therapy method that is portable, nontoxic, and easy to operate. Furthermore, the acupressure wrist-ankle strap is environmentally friendly due to its low-carbon characteristics and can be recycled.

If the proposed RCT can verify our hypothesis that the acupressure wrist-ankle strap is effective, it can be used as an immediate and effective physical intervention to relieve abdominal pain for patients with primary dysmenorrhea. The proposed RTC may also provide evidence for the analgesic effects of WAA and promote the application of WAA in clinical practice.

There exist some limitations of this proposed study. First, we will observe only the effects of one 30-minute treatment session, mainly focusing on the effect of 3 time points during the intervention period, for the main purpose of this study is to evaluate the immediate analgesic effect. Further work will be required to determine the longer-term effect of this device and whether this will be safe and acceptable for patients to use it frequently over several days. Second, the recruitment of participants is restricted to the students of a university with a great proportion of females, to include enough samples in a specific time period and to ensure good compliance of the participants. This will limit the external validity of the results. We will test the effects of this device with more diverse samples in the future study.

### 3.1 Trial status

Recruitment of participants started on May 1, 2019 and is anticipated to end on May 30, 2020. (The protocol is Version 1.0, dated April 10, 2019.)

## Author contributions

The trial was designed and developed by SJZ and QHZ. The manuscript was drafted by SJZ and YL. The protocol was critically revised and edited by YR, CX, and FFF for important intellectual content. ZL contributed to the sample size calculation and statistical analysis. SJZ, YR and QHZ contributed to the discussion. All authors read and approved the final manuscript.

Qing-hui Zhou orcid: 0000-0002-8772-994X.
